# How do Australian mental health services use easy read to make information accessible for people with intellectual disability?

**DOI:** 10.1111/jar.13156

**Published:** 2023-09-08

**Authors:** Bronwyn Newman, Karen R. Fisher, Julian Trollor

**Affiliations:** ^1^ Centre for Health Systems and Safety Research (CHSSR) Australian Institute of Health Innovation Macquarie University Sydney New South Wales Australia; ^2^ Social Policy Research Centre University of New South Wales Sydney New South Wales Australia; ^3^ Department of Disability and Neuropsychiatry University of New South Wales Sydney New South Wales Australia

**Keywords:** accessible information, easy read, intellectual disability, mental health

## Abstract

**Introduction:**

Access to health information is a right for all people. Easy read information is one strategy used to make information accessible for people with intellectual disability. This research explored how easy read is used and the ways accessible information can address access barriers, with a focus on Australian mental health services.

**Methods:**

Semi‐structured interviews were conducted across four sites to explore how easy read was used. Participants (*n =* 49) were easy read users, health practitioners and staff from services providing mental health care in New South Wales, Australia. An integrated health literacy framework was used to analyse data.

**Results:**

Most mental health staff did not use easy read or other accessible information, and did not consistently offer people with intellectual disability opportunities to understand, appraise and apply health information. This investigation confirmed the limited availability of accessible information resources, including easy read, and the importance of relationships of support when accessing health information.

**Conclusion:**

People with intellectual disability did not routinely have access to mental health information. Substantial change is required to address this disparity.

**Implications:**

Agency policy and processes require change to support staff practices that uphold the right to information. Inclusive practices that incorporate using easy read in health contexts, including mental health, are needed to facilitate change.

## 1 BACKGROUND

The right to accessible health information outlined in the United Nations Convention on the Rights of Persons with Disability (UNCRPD, 2006) is not always realised for people with intellectual disability. Cognitive accessibility of information, including health information, is a relatively recent consideration. The cultural shift away from dominant historical perceptions that information was not wanted or needed by people with intellectual disability is reflected in the UNCRPD, as the right to information is enshrined as a conduit for many other rights to be realised. The UNCRPD, of which Australia is a signatory, defines the right to health information in various formats, tailored to meet individual need. Easy read information is often advocated as an option for accessible information for people with intellectual disability despite limited evidence about how easy read is used.

The increasing commitment to providing information in accessible formats is reflected in a growing international policy commitment to meeting this obligation (Mcewin & Santow, 2018). In the United Kingdom for example, health services are required to provide accessible information, and hence have a substantial impetus to provide easy read. In Australia, the right to information access is expressed in policy, but the needs of people with intellectual disability are rarely articulated and formats such as easy read are not mandated (Dew et al., 2018; Newman, 2020; Reppermund et al., 2018). Recent recognition of the need for accessible information in the Australian health and mental health contexts is evident in the National Roadmap for Improving the Health of People with Intellectual Disability (Commonwealth of Australia, 2021) which provides guidance for health services across Australia, and the 2018 National Roundtable Communique which has specific guidance for mental health services (Department of Developmental Disability and Neuropsychiatry, 2018).

Increased availability of accessible health information is one strategy to address the longstanding disparities in health and mental health outcomes experienced by people with intellectual disability in Australia and internationally. People with intellectual disability experience poorer access to services and poorer health outcomes (Reppermund et al., 2020). Services for people with intellectual disability who experience mental ill‐health remain inadequate, complex and difficult to access in Australia and internationally (Lennox et al., 2015; Trollor, 2014; Venville et al., 2015; Whittle et al., 2019). Staff in mainstream services feels underequipped and unsupported to meet the needs of people with intellectual disability (Weise et al., 2017; Weise et al., 2020). Accessible information is a vital element in seeking to address this disparity as limited access to information and opportunity for engagement continues for many people with intellectual disability (Commonwealth of Australia, 2020; Department of Developmental Disability and Neuropsychiatry, 2018). The interim report of the recent Australian Commonwealth Royal Commission into Violence, Abuse and Neglect of people with disability illuminates the disparity in access to adequate health information and emphasises the need for increased staff awareness and training to support staff to communicate effectively with people with intellectual disability (Commonwealth of Australia, 2020, p. 20).

The personalised models of care central to many health and disability services are predicated upon service users having sufficient information to enable choice. Models such as recovery‐oriented practice and trauma informed care are central to mental health service delivery, and provide increasing opportunities for choices in care and support (Mental Healthcare Commission, 2014). Similarly, disability services have developed various person‐centred systems in accessible formats which facilitate inclusion of people with intellectual disability in both individual and agency‐wide planning processes (Gosse et al., 2017; Sanderson, 2014). The increasing shift toward personalisation in mental health, health and disability services reinforces the need for information access to enable people with intellectual disability to participate in fundamental processes.

Documents created to make information easier to understand for people with intellectual disability are often called Easy read or Easy English. Easy read use has increased despite little consistency in definitions of easy read, and little evidence about how easy read is created or used (Chinn & Homeyard, 2017; Sutherland & Isherwood, 2016). In this paper, easy read is defined as:

adapted written resources that are created for people with intellectual disabilities typically using short sentences, jargon‐free language and verbal text supported or explicated through visual images. (Chinn, 2020 p. 1)

Accessible information is one element, among many, in the health care context which impacts opportunities to learn and make decisions about health (Chinn, 2017). Tailored communication (Hemsley et al., 2012; Iacono et al., 2014), family and supporter involvement (Chinn, 2017; Hemsley et al., 2011; Mastebroek et al., 2016) and the healthcare provider relationship (Mastebroek et al., 2014; Mastebroek et al., 2016) have all been shown to affect opportunities for people with intellectual disability to use health information. Contemporary understandings of health literacy suggest a framework to incorporate these influences when exploring information access, including the central role of relationships of support as described in the concept of distributive health literacy (Geukes et al., 2019) According to Sorensen et al. (2012), health literacy:

entails people's knowledge, motivation and competences to access, understand, appraise, and apply health information in order to make judgements and take decisions in everyday life concerning healthcare, disease prevention and health promotion (Sorensen et al., 2012, p. 3).

An integrated framework *incorporating medical and public health approaches* to health literacy is used in this paper to scaffold the analysis using the concepts of accessing, understanding, appraising and applying information. These concepts are used to explore the experience of easy read use, with a focus on health service accessibility and capacity as described in emerging models of organisational health literacy (Trezona et al., 2017).

This qualitative research contributes new knowledge by using a health literacy framework to explore the research question:

How is easy read and accessible information used by staff working with people with intellectual disability in mental health services?

## 2 METHOD

### 2.1 Procedure

Appropriate ethical approval was obtained according to the National Health and Medical Research Council guidelines. Consent was gained from participants as per ethical guidelines and approvals. Researchers relied upon recruitment health agencies known to promote the research project and remained at ‘arms length’ to the process. Consent forms were available in accessible formats, completed with support from health agency staff when required and re‐confirmed at the time of interview. Data were collected, analysed and reported in accordance with COREQ guidelines.

Individual semi‐structured qualitative interviews were conducted by one researcher (BN) to explore staff and service user experiences of using easy read and other forms of accessible information if used (Supplementary document S2: interview schedule). The interview schedule was used as a guide to explore issues surrounding easy read availability and use. Thematic and interpretive approaches were used to analyse interviews using framework analysis to determine themes using concepts from the Integrated Health Literacy Framework (Sorensen et al., 2012).

Interviews were audio recorded with participant permission and transcribed verbatim. Interview participants included people with intellectual disability, their carers or supporters, nursing and allied health staff, medical practitioners and administrative staff. Interview lengths ranged from 9 to 56 min (Summary of interview participants Table 1). One person with intellectual disability preferred to communicate via a hand held speech to text device and one participant with intellectual disability chose to attend with a paid support worker. Three joint staff interviews were included and the data were analysed separately for each participant who attended a joint interview as the participants did not always express common views.

**TABLE 1:** Participant characteristics.

	No.	Interview time (minutes)	Role	Time in position
AGENCY 1	1	24	Psychiatrist	3 months
	2	Joint interview	Registrar	1 month
	3	29	Nursing manager	15 years
	4	Joint interview	NUM	6 years
	5	23	Psychologist	18 months
	6	25	Social worker	11–12 years
	7	20	NUM	6–7 years
	8	31	OT	18 months
AGENCY 2	9	33	Peer support worker	19 months
	10	52	OT/ID inclusion specialist	18 months
	11	42	Manager‐OT	16 months
	12	15	NUM‐inpatient	4.5 years
	13	9	NUM‐MHICU	3 months
	14	35	Social worker‐older people	Data not provided
	15	44	Psychiatrist	3 years
	16	42	Social worker‐older people	Approximately 30 years
	17	29	Psychologist	3 months
	18	20	Youth support/intake	2 years
	19	Joint interview	Youth support/intake	2 months
	20	Joint interview	Youth support/intake	2 months
	21	9	Manager	3.5 years
AGENCY 3	22	44	Paediatrician	6 months
	23	20	Registrar	5 months
	24	30	Parent/carer	10 years
	25	48	Dietician	9 months
	26	Joint interview	Exercise physiologist	6 months
	27	Joint interview	RN	6 years
	28	(approx. 35 min) Recorder malfunction	Evaluation of program	Data not provided
	29	Joint interview	Reception/admin	Data not provided
	30	19	Psychologist	7 years
	31	30	Psychologist	2 years
	32	16	Coordinator	8 years
	33	33	Psychologist	5 months
	34	20	Psychologist	8 years
	35	32	Psychologist	14 years
	36	48	OT	2 years
	37	56	Psychiatrist	10 years
AGENCY 4	38	24	Service user	9 years
	39	24	Service user	Data not provided
	40	Joint interview	Support worker	Data not provided
	41	14	Service user	10 years
	42	27	Service user	6 months
	43	8	Service user	Couple of years
	44	31	Service user	Data not provided
	45	26	Service user	1 year
	46	47	Advocate	4 months
	47	51	Manager—inclusion team	18 months
	48	53	Advocacy and support	2 years
	49	47	Participation worker	2 years

### 2.2 Recruitment

Purposive sampling was used to recruit participants via four agencies to enable data collection from a diverse range of participants. Agencies providing mental health services to people in a geographic region, including people with intellectual disability, were eligible for inclusion in the research, as well as specialised services for people with intellectual disability. Individual participants were eligible if they accessed services at one of the participating sites, or supported a person with intellectual disability to access the service. People with intellectual disability who used and/or created easy read were invited to take part. Individuals who were experiencing significant mental distress or psychosis were not eligible to participate. Agencies who did not offer support or information for people with intellectual disability were not approached to take part in this study.

### 2.3 Participants

Forty‐nine participants took part in semi‐structured interviews across four sites. The four sites comprised of one disability advocacy service, two mainstream public mental health services and one specialised intellectual disability public mental health service. All three mental health services were publicly funded and provided community, inpatient and outpatient care. The term ‘participants’ is used throughout this paper to refer to interview participants from all sites unless otherwise specified.

### 2.4 Data analysis

Data were analysed using iterative categorisation (Neale, 2016) to explore themes in relation to the integrated health literacy framework (Sorensen et al., 2012). Data were coded as it was collected by one researcher (BN), analysed in reference to all other agency data and crossed checked with a second researcher (KF). Once all coding was complete, findings were collated into a summary document. Themes and commonalities were identified and data were managed using NVivo 11 (QSR International Pty Ltd, 2015).

Iterative categorisation was used as it offers a rigorous, staged approach to analysing qualitative data across multiple sites and is appropriate for use with deductive studies with prescribed areas of interest (Neale, 2016). Adhering to the detailed, staged process outlined by Neale instilled greater confidence in the repeatability of the analysis process across multi‐site data collection.

## 3 RESULTS

Most mainstream mental health staff did not use easy read or other accessible information and did not consistently offer people with intellectual disability opportunities to interact with mental health information. The easy read resources described predominantly related to individual plans or care activities, for example exercise plans, strategies to reduce anxiety and tailored condition‐related information. Staff at specialised sites had numerous strategies they used to make information easier to understand, including easy read, and people with intellectual disability valued flexible, tailored approaches to communication. Participants emphasised that relationships between people with intellectual disability, family or carer and service providers significantly affected opportunities to interact with health information. The results are explored using the categories of accessing, understanding, appraising and applying information, as defined in the integrated health literacy framework devised by Sorensen et al. (2012).

*Access* is defined as the opportunity to ‘seek, find and obtain’ information (Sorensen et al., 2012, p. 9). In this research access relates to the availability of health information suitable for people with intellectual disability at health agencies. Availability of accessible resources such as easy read documents is vital, but did not guarantee that information access for all people. Four key elements were identified to facilitate *access* to information: availability, equipping staff, inclusivity and respectful relationships.

Information developed to suit the needs of people with intellectual disability, including easy read, was not consistently available at the participating sites. Most participating health service staff were aware of their responsibility to provide access to information but in many settings, did not have access to appropriate resources to provide or create accessible information Several participants at specialised sites created accessible versions of information for individual service users as required, many reinforced the benefit of tailored information. However, absence of ready‐made or tailorable resources made creating resources time consuming for individual staff and required a level of skill, confidence or expertise outside the capacity of many practitioners. This finding was reflected in the experiences of participants with intellectual disability who had not been offered easy read material in health service settings. Staff identified that easy read information was lacking in both specialised and mainstream services, reflected in comments such as:

We do that [provide information] as much as we can verbally, but often we don't have those information [written documents] to support that … we don't have that. (staff member from Agency 2)

The comments from staff about the availability of easy read resources were reflective of broader themes about the need for agency support to facilitate information access. Reflecting other recent research (Weise et al., 2017; Weise et al., 2020), many staff in mainstream mental health services did not feel confident about communicating with people with intellectual disability. Several staff participants expressed a need for increased support including, training or mentoring. Numerous staff in mental health services said that they felt ‘lucky’ to have access to a staff member who had previously worked with people with intellectual disability, or expressed gratitude to colleagues who supported them as an extra duty or act of kindness. Staff expressed a desire to provide accessible information and recognised that accessible health information was not often available to them. This sentiment was reflected in comments such as: ‘I don't think we are doing a lot but there is a lot of goodwill’ (staff from Agency 1).

Staff described the benefits of flexible practices to accommodate difference. Participants from all sites recognised barriers such as complex mandatory paperwork or forms and referral processes, and the impact of limited opportunities for consistent staff across appointments on information access for people with intellectual disability. Many staff said that the culture and pace of mental health services often precluded information access and to information. One doctor at Agency 1 commented: ‘At a busy clinic with performance targets it can be difficult to be friendly running a very tight ship’. Several participants raised concern about the impact of the health care environment, and the importance of staff awareness of the potential impact on communication:

There is a very high chance that the person/patient will agree with things or say yes to things even if they don't understand or don't agree … I think there is such a massive power imbalance… so being conscious of that is really important for health professionals. Staff member at Agency 4.

Participants from all sites highlighted the importance of inclusive, welcoming environments to facilitate relationships between people with intellectual disability, health service providers and carers/families to enable access to information. Respectful relationships with family and supporters, and health service staff facilitate advocacy for appropriate resources, tailoring information content and format. A parent interviewed from Agency 3 often attended appointments on her son's behalf and relayed the weight of responsibility as the sole conduit for health information. For other participants with intellectual disability, family and supporters provided emotional support, were a sounding board for discussion about health‐related decisions or for others, family members were the source of persistent reminders about health checks. Many staff valued the input of family and supporters to provide suitable communication tools, such as easy read or pictorial communication strategies. However, some staff raised the concern that their reliance on family to facilitate communication could mask systemic inadequacies to meet the needs of people with intellectual disability.

*Understanding* or comprehending information is next phase in Sorensen et al's integrated health literacy model. The data within this theme related to the practicalities of easy read development and comprehension, but most significantly to the interaction between health practitioners, service users and supporters (see Supplementary File S1 for Coding tree). Participants who used easy read were unanimous in their view that easy read facilitated greater understanding. Easy read users valued the pictures as a prompt for conversation and the terminology as a common language to use across conversations. Four participants with intellectual disability valued easy read to increase their opportunities to understand information independently, however for most people, easy read was used with support. Easy read was used to facilitate conversations and provide a context for asking questions. One health practitioner from Agency 1 said that it was to read together ‘Not just give this [easy read document] and goodbye’. Easy read was identified as a valuable tool to create a shared vocabulary between people with intellectual disability and their supporters, including health staff. Staff identified that having easy read available gave them greater confidence to pitch information appropriately.

Staff across all sites emphasised the need to ‘know the patient’ to enable them to select the most suitable mode of information delivery. Tailoring information to facilitate understanding was dependent on establishing relationships with people with disability and their families/supporters, in tandem with staff confidence and skill to adapt information. Staff experienced in working with people with intellectual disability said it was important to ‘follow the person with intellectual disability's lead’, and this required a knowledge of the person's preferences and support networks, as well as the communication strategies available. Staff who had used accessible resources implemented various communication strategies to enhance understanding and reinforced that easy read was one of many strategies required to tailor information.

Participants familiar with easy read said that using accessible information, including easy read, had benefit for both service users with intellectual disability and health practitioners. Easy read users said that it enhanced both the understanding of the person with intellectual disability of health information, and the understanding of the health practitioner of the needs and preferences of the person with intellectual disability. One participant with intellectual disability from Agency 4 spoke about using easy read to facilitate communication at appointments and the nature of communication: ‘Yes it's a two way thing … it's not a one way thing … it also can go to three ways if you take someone with you’. A specialist disability support worker at a Agency 2 identified the impact of the interplay between building relationships, the availability of accessible information and staff expectation of people with intellectual disability:

Sometimes there is a preconception that people [with intellectual disability] cannot understand but the problem may be that you [Agency staff] are not giving the information in a format that is easy to understand.

*Appraise* is defined as ‘to interpret, filter, judge’ information and *applying* is using the information accessed and understood. The themes identified in interview data about *appraising* mirrored themes for *applying i*nformation and have been presented together. In this research, the opportunities to *appraise and apply* information presented in interviews were at times immediate (eg. decisions about daily care), but more often related to activities to undertake away from an interaction with health practitioners (e.g., Exercise programmes or medication regimes). Three key benefits of easy read or accessible information were identified: facilitating interaction, creating expectation of involvement and increasing confidence of both staff and people with intellectual disability to engage.

People who use easy read said that the simple text and pictures provide a shared vocabulary and common understanding which enabled communication between people with intellectual disability and multiple practitioners and supporters when applying information. This was seen in a limited way in mainstream inpatient facilities where easy read was most often used to maintain calm, by informing patients of routines or expectations to *apply* by making immediate choices. Staff noticed an increased level of compliance and less distress for people with intellectual disability in these settings when easy read was available. Whereas services that routinely included people with intellectual disability used accessible information and easy read to inform decision making and plan collaboratively. Staff who used accessible resources said that they are effective for activities such as exercise plans and anxiety reduction strategies. One participant with intellectual disability discussed the benefits of tailored, easy read instructions for administering her medication, particularly when travelling overseas. This level of engagement to inform choice and plan treatment was not consistently evident in mainstream services.

Staff who used accessible information to facilitate interaction saw that easy read signalled an expectation of inclusion. Providing simplified text and relevant pictures set the expectation that the interaction would proceed with an inclusive approach. One staff member from Agency 2 commented that easy read information: ‘Sets the tone and acts as a reminder in the relationship’. The expectations or assumptions of health staff, supporters and family about the willingness or ability of a person with intellectual disability to discuss health information impacted opportunities to make related decisions and implement associated actions. One supporter from Agency 4 said ‘they [family] do things for them because they think they [the person with intellectual disability] can't do them for themselves.’ Another staff member at Agency 2 commented about her workplace ‘currently the underlying assumption is that they [people with intellectual disability] don't have the ability to make good choices’.

Several staff said that easy read information helps them and other supporters to develop an understanding about what the person with intellectual disability understands, wants or is concerned about, which was particularly important in appraising options. Using a simplified version of complex terms enabled staff to communicate complicated medical terms or treatment more confidently. Many participants raised concern that a lack of accessible information limited opportunities for people to question issues or interact when considering options. A supporter of people with intellectual disability at Agency 2 said:

I think in a lot of situations in health it's[decision about care] just pushed through and support people make decisions for them sadly – because there's that time pressure then there is a lack of information.

Participants who used easy read and other accessible communication strategies saw that they are an essential component in addressing the engrained communication constructs evident in the health service environment. At all sites, staff expressed a need for more accessible resources, and significant changes to agency practices, to enable people with intellectual disability to appraise and apply information.

## 4 DISCUSSION

This research showed that the participating mental health agencies did not consistently provide accessible mental health information to people with intellectual disability. Only the agencies specialised in working with people with intellectual disability used accessible information and easy read about mental health. Participants with intellectual disability were unanimous about the benefits of easy read, but had not accessed easy read when attending health services. Mental health staff were not routinely supported by guidelines or training about how to communicate clearly with people with intellectual disability, had minimal accessible information available to them and most had not used easy read. Most mental health staff did not think people with intellectual disability were provided adequate information or time to make decisions about mental health information. Easy read or other tailored accessible resources were essential, but one element of many required to make mental health information accessible. Having enough time to consider information and respectful relationships with health practitioners and other supporters was vital for people with intellectual disability interacting with health information. The availability of easy read signified that people with intellectual disability have a right to information, but was not a guarantee of this right being realised.

Using the concept of *access* in this research further underlined agency responsibility to provide tailored information, and exposed the mismatch between what people with intellectual disability need and what the system provides. The participants of this research provided insight into a system not built for difference or equipped to tailor information to meet individual need. The international experience, particularly the UK, has confirmed that availability of easy read formats is not sufficient to guarantee access to information for all people, but is a vital first step (Kean, 2016). Significantly, this research has exposed that the Australian agencies explored in this study do not have this first step available to staff. These findings further illuminate issues raised in various forums within Australia (Commonwealth of Australia, 2020; Department of Developmental Disability and Neuropsychiatry, 2018) and internationally (Foundation for People with Learning Disabilities, 2014), highlighting that flexible approaches to facilitate opportunities for support are crucial, yet currently absent.

The use of accessible information resources, such as easy read, within relationships of support to *appraise and apply* information further develops recent health literacy research and theory development. These findings reinforce previous studies which have established the potential for people with intellectual disability to be excluded from decision making when accessible information is overlooked, reinforcing the need for practice change (Watson, 2016; Watson et al., 2022). The need for increased research about the distributive nature of health literacy across relationships of significance in the lives of people with intellectual disability has been recognised, and this research goes some way in exploring this gap (Geukes et al., 2019). Using the health literacy framework to structure this research illuminates and confirms the relationship between information access, and opportunities to appraise and apply health information within relationships of support.

The importance of respectful relationships of support between people with intellectual disability, family, health care practitioners seen in this research reinforces the need for health service environments to foster such relationships. Studies in Australia and internationally have demonstrated the impact of the health care context on communication (Chinn, 2017; Iacono et al., 2020). Researchers and practitioners have called for systemic change to address healthcare structures that limit access to information for people with intellectual disability (Chinn & Rudall, 2021; Iacono et al., 2020; Kean, 2016; Newman, 2020). There are a multiplicity of factors to address to create more inclusive services including; development of policy to foster a culture of inclusion by endorsing flexible practices, making appropriate communication strategies readily available and providing staff training and mentoring (Weise et al., 2017; Weise et al., 2020).

## 5 STRENGTHS AND LIMITATIONS

The varied views expressed by participants, their generosity and frankness were strengths of this research. Using the integrated health literacy framework provided a valuable pathway to incorporate intellectual disability into broader considerations about health service access and outcomes. The health literacy framework provided a scaffold for investigation, potential limitations in using a framework approach were minimised in the incorporation of iterative categorisation methodologies (Neale, 2016).

Limitations of this research primarily relate to the availability of easy read mental health information at the included sites. Easy read information was used rarely in the participating mental health settings. This finding is significant, yet also limits research about easy read in the mental health service context. The second limitation is that although ethical approval was obtained to interview people with intellectual disability accessing participating services, no suitable participants were available within the research timeframe. This limitation was not unexpected due to the nature of mental health services engaged in the research and was managed by including people with intellectual disability who were eligible to access mental health services, made and used easy read. Similarly, the inclusion of the views of carers or supporters was invited, but their input was limited.

## 6 IMPLICATIONS

Significant changes are required for access to information about mental health by people with intellectual disability. Systemic change is required to support access to information as a right not a favour. Researchers, policy makers and advocates have called for increased availability of accessible resources and support for staff to routinely use inclusive practices (Commonwealth of Australia, 2021; Department of Developmental Disability and Neuropsychiatry, 2018). Mandating the collaborative creation of easy read information is a first step, but not sufficient alone. Policy change is required to enable the development of a culture of inclusion, with flexible practices, opportunities for relationships of support to be fostered and staff supported to implement a diverse array of communication options.

## FUNDING INFORMATION

This study was funded by a National Health and Medical Research Council Australia funded Partnerships for Better Health grant (ID: APP1056128; Title: Improving the Mental Health Outcomes of People with an Intellectual Disability).

## Supporting information


**Data S1.** Supporting information.

**Data S2.** Supporting information.

## Data Availability

Data available on request due to privacy/ethical restrictions.
