# Supplementary figures and images for: How do Australian mental health services use easy read to make information accessible for people with intellectual disability?
Source: J Appl Res Intellect Disabil. 2023 Sep 8;36(6):1354–62. doi: 10.1111/jar.13156 (PMC10946455; doi:10.1111/jar.13156)

SUPPLEMENTARY FILE : Coding tree


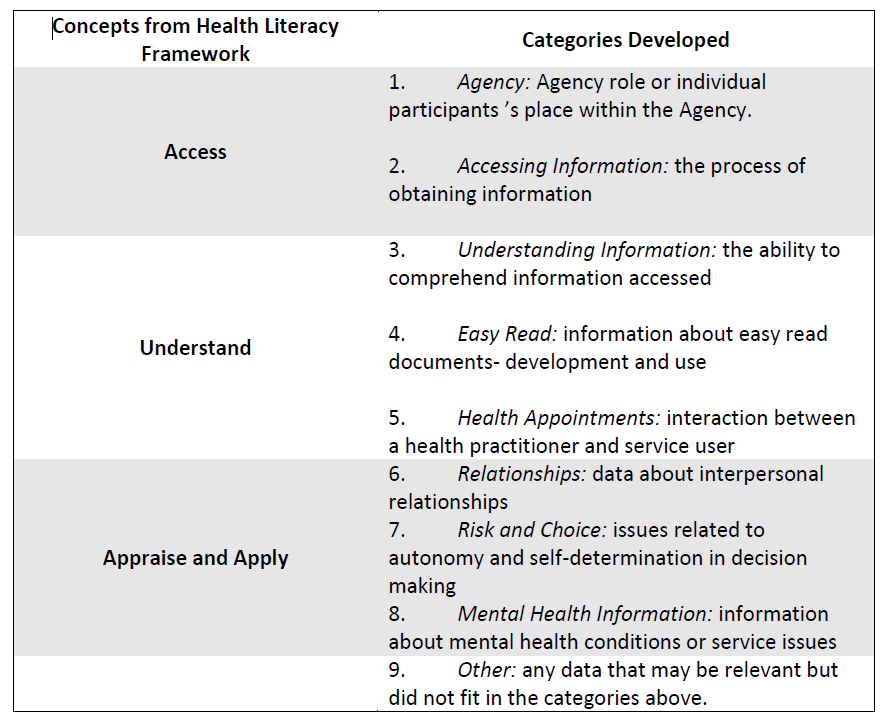

Supplement: Supplementary file 1 — Data S1. Supporting information. [file JAR-36-1354-s002.docx]
