# Supplementary material for: How do Australian mental health services use easy read to make information accessible for people with intellectual disability?
Source: J Appl Res Intellect Disabil. 2023 Sep 8;36(6):1354–62. doi: 10.1111/jar.13156 (PMC10946455; doi:10.1111/jar.13156)
Supplement: Supplementary file 2 — Data S2. Supporting information. [file JAR-36-1354-s001.docx]

**SERVICE PROVIDER INTERVIEW QUESTION GUIDE - HOW CAN ‘EASY READ’ MAKE INFORMATION**

**ABOUT MENTAL HEALTH MORE ACCESSIBLE FOR PEOPLE WITH INTELLECTUAL DISABILITY?**

| **QUESTION GUIDE FOR SERVICE PROVIDERS** | | |
| --- | --- | --- |
| 1. What is your primary role at [SERVICE] How long have you worked at [SERVICE]? | | |
| 1. Do you work directly with people who have intellectual disability and their families? | | |
| 1. In your experience, what are the most important factors in making information understandable for someone with intellectual disability? *(explore themes as they arise- tailored information, family carer, awareness of power differential*) | | |
| 1. I am interested to hear how this relates to conveying health information? *explore particular considerations for people with intellectual disability who experience mental ill-health* | | |
| 1. Are you familiar with the term ‘easy read’? | | |
|  | YES (5a-5d) | NO (5e-5h) |
|  | 1. At your agency what constitutes an ‘easy read’ document? | e. Show an example |
|  | 1. Where do you access these documents?   If they produce their own …what guides the layout and design? | f. Is this something you use? |
|  | 1. How do you use ‘easy read’? (*give to person with intellectual disability,read through it /facilitation of information appraisal- exploration of options?)* | g. what kinds of techniques /tools do you use to enable access to information? |
|  | 1. What kinds of subjects are covered in ‘easy read’ format ? (*eg tailored health info,service guides?)*   *Have you had experience in providing information about mental health in an ‘easy read’ format?* | h. How do these techniques/tools enhance understanding? |
| 1. Are there other things that you see your agency does well to make information for people with intellectual disability i) available?   And ii) understandable?  *And* *Not so well?* | | |
| 1. I am also interested in the way that accessible information provides opportunity for people with intellectual disability to explore options for service or treatment- are there ways that your service encourages this? *Explore appraise/apply in relation to agency role,link to strategies discussed in previous questions* | | |
| 1. How do you see that the policy and procedures that govern the work at CID impacts information accessibility? | | |
| 1. Is there anything else about making information accessible for people with intellectual disability that you would like to share? | | |

Questions below will be used as a guide and tailored according to interview tone and responses. General introductions to one another and to the aims of the project, housekeeping and warm up will be undertaken prior to questions as appropriate.

**SERVICE USER QUESTION GUIDE- HOW CAN ‘EASY READ’ MAKE INFORMATION ABOUT MENTAL HEALTH MORE ACCESSIBLE FOR PEOPLE WITH INTELLECTUAL DISABILITY?**

| SERVICE USER QUESTION GUIDE (people with intellectual disability and their carers) | | |
| --- | --- | --- |
| 1. Who do you come to see at [SERVICE] | | |
| 1. How often do you see them? 2. When did you first come? 3. What do you usually do when you come to [SERVICE]? 4. Do you usually bring [CARER] with you or\ come alone? | | |
| 1. I would like to learn more about the ways that you get information -I am especially interested in information about health. How do you usually find out about health or health services? (*prompt if needed : Can you tell me about a ‘health appointment’ or whatever they describe as where they get information… that went well?* ) | | |
| 1. If someone is telling you something important or giving you information, how do you like them to tell you? In words,on paper? Are you the kind of person who likes to ask questions and talk about different options (*explore role of family/carer and opportunity to appraise information*) | | |
| 1. Do you ever talk to anyone or get information about ‘mental health’? *(explore – maybe its talking about how you are feeling or your worries?) Have you ever been given information about these kinds of things? Can you tell me about that?* | | |
| 1. Have you heard of ‘easy read’? | | |
|  | YES | NO |
|  | Show an example – is this what you think of? If no- what | Show an example |
|  | Where have you seen ‘easy read’? | Is this something you have ever seen used? |
|  | How did you use ‘easy read’? (g*iven to carer,read through it at Dr,took it home)* | Do you think it would be useful ? what for? |
|  | What was the ‘easy read’ about ? (*eg tailored health info,service guides?)* | Relate to previous discussion – how they access, understand information-clarify |
| 1. I am planning to write a report that will go to places like doctors and hospitals /people who decide how information is given out or talked about– so I want to know if there is anything you would like to say to them. So I want to ask-   What is the most important thing for people to do when they give you information about your health or being healthy? (*re-phrase & prompt* *to focus on* *mental health if appropriate*) | | |
| 1. (if undisclosed in interview) Just as we finish would you mind if I ask for your…   Postcode Age range 18-25,25-35,35-45,45-55,55 +  Living arrangements –independent, family, group home style accommodation  Service usage history | | |

The language for each question has not yet been established as it will be dependent on the needs of the individual and tone of the interview. The questions schedule is designed to be as flexible as possible so as to allow the interviews to be steered by participants.

The interview will commence with confirmation of consent /General introductions to one another and the aims of the project, housekeeping and warm up questions.
